# Oocyte related factors impacting on embryo quality: relevance for *in vitro* embryo production

**DOI:** 10.21451/1984-3143-AR2018-0077

**Published:** 2018-08-17

**Authors:** Fabienne Nuttinck

**Affiliations:** 1 Université Paris Saclay, Jouy en Josas, 78350, France.

**Keywords:** cumulus cells, germinal-somatic interactions, maternal RNAs, oocyte maturation, preimplantation development

## Abstract

The outcome of pregnancy is closely linked to early events that occur during the onset of embryogenesis. The first stages in embryonic development are mainly governed by the storage of maternal factors present in the oocyte at the time of fertilisation. In this review, we outline the different classes of oocyte transcripts that may be involved in activation of the embryonic genome as well as those associated with epigenetic reprogramming, imprinting maintenance or the control of transposon mobilisation during preimplantation development. We also report the influence of cumulus-oocyte crosstalk during the maturation process on the oocyte transcriptome and how *in vitro* procedures can affect these interactions.

## Introduction

The notion of embryo quality refers to the capacity of an embryo to develop and support successful pregnancy to full term. In cattle, most failed pregnancies result from embryonic mortality that occurs before implantation, during the first two weeks after fertilisation ( Berg *et al*., 2010 ; Lonergan *et al*., 2016 ). During this window, the fertilised oocyte undergoes profound morphological changes. Embryonic cell divisions and cavitation lead to blastocyst formation. The blastomeres segregate between the inner cell mass and trophectoderm which gives rise to initiation of the first lineages, namely the embryonic epiblast, extraembryonic hypoblast and trophoblast ( Artus and Chazaud, 2014 ). The embryonic epiblast will give rise to all future intraembryonic tissues while extraembryonic tissues will be involved in forming the placenta ( Ribeiro *et al*., 2016 ; Schroder *et al*., 2016 ). Cellular differentiation and lineage specification require prior resetting of the epigenetic information that is contained in each gamete to enable a return to totipotency. It also necessitates processes to restrict further expression to lineage-appropriate subsets of genes. Epigenetic information participates in mechanisms that preside over genetic information. This consists in a combination of marks such as DNA methylation and posttranslational histone tail modifications ( Kouzarides, 2007 ). The extensive modifications of parental epigenetic marks that occur soon after fertilisation and are referred to as early epigenetic reprograming are involved in regulating gene expression throughout early development ( Canovas and Ross 2016 ; Sepulveda-Rincon *et al*., 2016 ; Zheng *et al*., 2016 ). A small fraction of genes within the whole genome escapes from the extensive DNA methylation erasure that occurs in the zygote. These genes include imprinted genes that display different types of DNA methylation depending on their parental origin, leading to parental-allele-specific gene expression during development. Genome imprinting plays a key role in maintaining normal embryogenesis ( Elhamamsy, 2017 ). In cattle, genomic reprogramming has been shown to be associated with an increase in the expression of transposable elements (TEs), specifically from a subclass of these elements called retrotransposons ( Bui *et al*., 2009 ). Because the potential proliferation of TEs within the genome due to autonomous copy-and-paste mechanisms can result in genome instability and may damage the embryo, this transposon mobilisation needs to be constrained to permit normal embryo development.

Mammalian embryos are transcriptionally quiescent at the start of development. Early embryogenesis is mainly governed by post-transcriptional and post-translational events. The stockpile of maternal RNAs and proteins, which are stored within the oocyte during oogenesis, sustains the first stages of development until embryonic genome activation (EGA; Vigneault *et al*., 2009 ; Deutsch *et al*., 2014 ). Inherited factors from the oocyte contribute to epigenetic reprogramming and imprinting maintenance during early development ( Canovas and Ross, 2016 ; Lodde *et al*., 2017 ). In cattle, factors that are likely to be involved in protecting the embryo against the deleterious effects of transposon mobilisation have been evidenced in the oocyte at the time of fertilisation ( Russell *et al*., 2017 ). The time frame for the embryo transcription machinery to be fully functional varies between species ( Telford *et al*., 1990 ). In cattle and sheep, the major EGA that conveys the transition from maternal to the embryonic control of embryo development occurs gradually from the 8- to the 16-cell stage ( Graf *et al*., 2014 ). This process is considered to be one of the most critical events governing bovine embryo viability during preimplantation development and is closely associated with the nuclear and cytoplasmic characteristics of oocytes at the time of fertilisation ( Gad *et al*., 2012 ).

Throughout this manuscript, we shall focus on several aspects of the oocyte transcriptome at the time of fertilization that are known to be related to a successful course of preimplantation development, and particularly with the achievement of EGA. We shall also discuss how cumulus-oocyte interactions can refine the oocyte transcriptome during the maturation process and how *in vitro* procedures may potentially affect these interactions and subsequent embryonic development in cattle.

## Impact of oocyte transcriptome on early embryo development

The storage of maternal RNA enclosed in the fertilised oocyte is involved in the control of early embryogenesis ( Tadros and Lipshitz, 2009 ). In cattle, several maternal transcripts (also known as maternal effect genes) have been shown to play an essential role during the first embryonic cleavage cycles, activation of the embryonic transcription machinery, pluripotency gene expression and blastocyst cell allocation ( Bettegowda *et al*., 2007 ; Tejomurtula *et al*., 2009 ; Tripurani *et al*., 2011a ). During oogenesis, the oocyte actively synthetises and accumulates a large collection of coding and non-coding RNAs ( Susor *et al*., 2016 ). Oocyte transcription gradually decreases during terminal differentiation within the preovulatory follicle, ceasing finally with the resumption of meiosis. Maternal RNAs are stabilised and stored so that they will be available over a period of several days that include embryo transcription silencing. RNA stabilisation mechanisms such as the de-adenylation and capping processes have been shown to control the translation of several key mRNAs in embryos in a timely manner ( Kim and Richter 2007 ; Richter, 2007 ; Weill *et al*., 2012 ). The extent of maternal transcript polyadenylation is positively correlated with translation efficiency. For example, JY-1, a maternal effect factor identified in the bovine oocyte, is required to reach the embryonic 8- to 16-cell stage and complete EGA ( Bettegowda *et al*., 2007 ; Lee *et al*., 2014 ). JY-1 transcripts display temporal variations in their adenylation status throughout the initial cleavages. The abundance of polyadenylated JY-1 mRNA is low during oocyte maturation; it increases at the pronuclear and 4-cell stages, and then decreases to almost undetectable levels after the 16-cell stage of embryo development. In contrast, the amount of total (de-adenylated + polyadenylated) JY-1 mRNA, which is highest in the immature oocyte, gradually decreases to become undetectable after the 16-cell stage. These changes to transcript adenylation lead to dynamically regulated JY-1 mRNA translation during early development.

Deadenylation and readenylation mechanisms are not always involved in regulating mRNA translation. The expression of Jumonji domain-containing protein 3 (JMJD3) during the early development of bovine embryos is a paradigm. JMJD3 belongs to the Jumonji family of genes that are epigenetic regulators. JMJD3 is a lysine demethylase associated with histone demethylation. Its activity is required for the removal of trimethylated histone 3 lysine 27 (H3K27me3) marks during the reprogramming process ( Canovas *et al*., 2012 ). The histone H3K27me3 mark is involved in the silencing of gene expression. At fertilisation, H3K27me3 marks have to be removed from the gametic chromatin in order to reactivate silenced genes, thus enabling EGA and development to the blastocyst stage ( Bogliotti and Ross, 2012 ). The level of JMJD3 mRNA, which is high in MII-stage oocytes, decreases from the zygote to the 16-cell stage. JMJD3 protein, which is undetectable in the oocyte, is translated soon after fertilisation and persists throughout the first cleavage cycles. Although JMJD3 protein expression is dynamically regulated during the window from fertilization to EGA, maternally inherited JMJD3 mRNA do not display changes to patterns of transcript abundance between the total and polyadenylated fractions, suggesting that other mechanisms are involved in the regulation of mRNA translation ( Canovas *et al*., 2012 ).

While tight temporal activation of the translation of dormant maternal mRNAs is required for successful progress through the first cleavages, destabilisation and degradation of the maternal RNA pool is a major determinant for the start of EGA ( Tesfaye *et al*., 2017 ). Inherited maternal micro RNAs (miRNAs) seem to be critical players in the control of maternal transcripts during early development in cattle ( Mondou *et al*., 2012 ). miRNAs are a large class of small non-coding RNAs (less than 200 nucleotides) that play important gene-regulatory roles in repression of the mRNAs of protein-coding genes ( Guo *et al*., 2010 ). miRNAs derive from long primary microRNA transcripts that are successively processed by the nuclear microprocessor complex Drosha-DGCR8 and by the cytoplasmic RNAse III enzyme Dicer. miRNA-mediated repression leads to a reduction in translational efficiency and/or decreased mRNA levels ( Dallaire and Simard, 2016 ). Decreased mRNA levels are associated with the mRNA de-adenylation and de-capping processes that trigger mRNA destabilisation and degradation. For example, NOBOX (Newborn ovary homeobox gene) is a maternal-derived transcription factor that is stage-specifically expressed during oocyte maturation and early embryonic development in cattle ( Tripurani *et al*., 2011a ). NOBOX is required for embryonic development to the blastocyst stage. It is involved in regulating POU5FI/OCT4 and NANOG pluripotency gene expression, blastocyst cell allocation and embryonic transcriptional activity. The 3’-untranslated region (3’-UTR) sequence of NOBOX mRNA exhibits a binding site for miR-196a. After fertilisation, NOBOX mRNA and protein expression are gradually suppressed from the 2-cell to 8-cell stages as the expression of mature miR-196a increases ( Tripurani *et al*., 2011b ). The highest abundance of mature miR-196a near the 8-cell stage of embryogenesis supports its involvement in maternal NOBOX mRNA degradation at the onset of EGA in cattle.

Piwi-interacting RNAs (piRNAs), another class of small non coding RNAs, are potentially involved in the control of maternal mRNA translation and decay as well as in that of transposon activity during early embryogenesis ( Barckmann and Simonelig, 2013 ; Russell *et al*., 2017 ; Zhang *et al*., 2017 ). These piRNAs associate with PIWI proteins to form ribonucleoprotein complexes referred to as piRNA-induced silencing complexes (piRISCs), which bind to RNA targets with complementary nucleotide sequences, leading to splicing activity, transcriptional repression and/or degradation. A recent study using bovine, macaque, and human material showed that most small RNAs that are present into MII-stage oocytes are represented by a piRNA-like population (approximately 26 nucleotides in length) whereas miRNAs account for less than 1% ( Roovers *et al*., 2015 ). A pool of piRNAs similar to that observed in oocytes can still be detected at the 2- to 4-cell-stage in bovine embryos. Testis-derived piRNAs with a length preference of approximately 30 nucleotides are not obvious after fertilisation, suggesting that embryonic piRNAs mainly derive from the oocyte. The oocyte-derived piRNAs pool is strongly enriched with transposon-derived sequences and may help to prevent transposon activity during genome reprogramming in cattle ( Russell *et al*., 2017 ). To be functional, the PIWI pathway requires the presence of both piRNAs and their associated PIWI proteins. A recent study in cattle evidenced the dynamically regulated expression of a transcript coding for the PIWI protein PIWI1during early development ( Russell *et al*., 2016 ). PIWI1mRNA expression displays a peak at the 2-cell stage, after which the levels fall through to the blastocyst stage. The PIWI1 mRNA expression profile, as well as the ability of PIWI1 protein to bind piRNAs, suggests its involvement in transposon control during embryonic reprogramming.

Long non-coding RNAs (lncRNAs) are increasingly being recognised as modulators of gene expression. These lncRNAs are a class of transcripts longer than 200 nucleotides that do not usually code for a protein ( Ruiz-Orera *et al*., 2014 ; Mattick and Rinn, 2015 ). Recent studies in mammals suggested functional roles for maternal lncRNAs during early embryonic development ( Taylor *et al*., 2015 ; Bouckenheimer *et al*., 2016 ; Svoboda, 2017 ). A transcriptomic analysis performed in human embryos evidenced the dynamic expression of oocyte-inherited lncRNAs which included TUBB8P7 (tubulin beta 8 class VIII pseudogene 7), BCAR4 (breast cancer anti-oestrogen resistance 4), WEE2-AS1 (WEE2 antisense RNA 1) and TUNAR (TCL1 upstream neural differentiation-associated RNA) during preimplantation development ( Bouckenheimer *et al*., 2018 ). Their expression remains stable during the first two cleavages. It then declines during progression to the blastocyst stage, specifically between the 4-cell and 8-cell stage, suggesting a role in embryonic cell division as well as in the control of gene activation at the onset of EGA. The clearance of lncRNAs may involve the post-transcriptional adenylation process ( Mattick and Rinn, 2015 ). A recent review reported the involvement of several lncRNA in control of the mono-allelic expression of imprinted genes at almost all stages of mammalian development, including preimplantation stages ( Saha *et al*., 2017 ).

## Effect of periconceptional cumulus-oocyte crosstalk on the oocyte transcriptome

The capacity of the fertilised egg to support successful embryonic development is reliant upon the content of the oocyte at the time of fertilisation. The phenotype of a mature oocyte is the culmination of continuous and highly coordinated interactions between the germinal and somatic compartments of the ovarian follicle that occur throughout folliculogenesis, and particularly during terminal differentiation of the cumulus-oocyte complex (COC) ( Li and Albertini, 2013 ). The LH surge induces cell signalling cascades within the preovulatory follicle leading to oocyte maturation, cumulus expansion and ovulation of the COC. Oocyte maturation consists of a stream of events that occur at both the nuclear and cytoplasmic levels and are often referred to as meiotic and cytoplasmic maturation ( Sirard *et al*., 2006 ). While the oocyte resumes meiosis and progresses to the metaphase II (MII) stage, cytoplasmic organelles (including mitochondria, endoplasmic reticulum and the Golgi apparatus) undergo important changes affecting their structure, function and/or distribution ( May-Panloup *et al*., 2007 ). There is a growing body of studies that have provided molecular explanations (concerning oocyte-somatic cell communication) for the underlying mechanisms of several cytoplasmic maturation features ( Cakmak *et al*., 2016 ; Sousa Martins *et al*., 2016 ). Epidermal growth factor (EGF)-like peptides have been shown to play a central role in transmission of the preovulatory LH signal from mural granulosa cells to cumulus cells (CCs) in several mammalian species, including humans ( Prochazka *et al*., 2017 ; Richani and Gilchrist, 2018 ). In turn, CCs exchange signals with the oocyte enabling both of them to reciprocally modulate the transcriptional and/or translational events that occur in preparation for fertilisation ( Conti *et al*., 2012 ; Conti and Franciosi, 2018 ). In mice, the interaction between EGF-like growth factors and cumulus EGFRs leads to activation of the phosphatityl-inositol-3-kinase-AKT-mechanistic target of rapamycin (PI3-AKT-mTOR) pathway in the oocyte. The mTOR signalling pathway is involved in controlling the translation of several maternal transcripts that are critical to embryonic development ( Chen *et al*., 2013 ). A recent study in mice showed that mTOR-dependent pathways in growing oocytes are also involved in the control of oocyte translation during the maturation process ( Guo *et al*., 2018 ). Up-regulated expression of the prostaglandin G/H synthase-2 (PTGS2) pathway in granulosa cells and cumulus cells following the LH signal has been reported in several mammalian species, including bovines ( Sirois, 1994 ; Nuttinck *et al*., 2002 ). Prostaglandin E2 (PGE2) is the main PTGS2-related prostaglandin secreted by follicular somatic cells. Autocrine and paracrine PGE2 signalling is required to intensify and propagate the EGF-like peptide signal within the preovulatory follicle ( Shimada *et al*., 2016 ). In turn, EGF-like peptides produced by CCs promote the mRNA expression of genes such as PTGS2 that are involved in the cumulus expansion process. Bovine oocytes express PGE2 binding sites, suggesting that PGE2 is involved in cumulus-oocyte coupling in cattle ( Nuttinck *et al*., 2011 ). The modalities of information exchanges between somatic and germinal compartments of the COC vary as oocyte maturation progresses ( McGinnis *et al*., 2013 ). Some hours before ovulation, gap junction communications between the oocyte and CCs are promptly down-regulated while the pattern of oocyte secreted factors continues to be regulated dynamically by feedback loops between the gamete and expanding CCs ( Coticchio *et al*., 2015 ; Cakmak *et al*., 2016 ).

Novel mediators of the intercellular communication that is involved in building the oocyte transcriptome during the preovulatory period have recently been highlighted. Studies in cattle have revealed that cumulus cells contribute to oocyte transcript stores by way of active RNA transfer. Before the initiation of meiosis resumes, large cargos including mRNAs and lncRNAs appear to move from the CCs to the oocyte through transzonal projections (TZP; Macaulay *et al*., 2014 , 2016 ). The abundance of several specific transcripts such as RASL11B (RAS like family11 member B), KIF5B (kinesin family member 5B) and AFF4 (AF4/FMR2 family member 4) mRNAs increases in the oocyte during transition from the immature to the mature stage, while endogenous transcription of the oocyte becomes silenced. In bovines, the potential for large cargo transfer from the CCs to the oocyte via TZPs terminates around 9 h after the induction of meiosis resumption when both compartments of the COC gradually dissociate and CC projections are released. This exogenous source of RNA may potentially enrich the pool of maternal factors that are involved in the control of early embryogenesis. It has been suggested that other classes of cumulus transcripts may be exchanged with the oocyte. The transcriptional profiles of human MII oocytes and their surrounding CCs indicate that many genes expressed in oocytes are potential targets of CC miRNAs, suggesting that oocyte-CC crosstalk might also be mediated via miRNAs ( Assou *et al*., 2013 ). Among the oocyte mRNA targets of CC miRNAs are transcripts coding for factors associated with chromatin remodelling such as the DNA methyltransferases DNMT1, DNMT3A, DNMT3B and SMARCA5 (SWI/SNF related, matrix associated, actin regulator of chromatin, subfamily a, member 5). An *in vitro* study using bovine model shown that modulation of miRNA-130b expression in maturing oocyte affects the meiosis progression as well as the proliferation rate and the glucose metabolism activity of surrounding CCs ( Sinha *et al*., 2017 ).

## The *in vitro* maturation process may alter cumulus-oocyte crosstalk

In cattle, *in vitro* embryos are produced through successive steps which include *in vitro* maturation (IVM) and fertilisation (IVF). The resulting zygotes undergo a 7-day culture period that permits them to reach the blastocyst stage. Embryo quality is assessed morphologically at the end of this culture period in order to select embryos that are compatible with the transfer procedure ( Rocha *et al*., 2016 ). IVM implies that the COC achieves its terminal differentiation in the absence of a follicular environment, and thus when LH-induced granulosa cell cues are absent. While nuclear oocyte maturation appears to be progressing normally, other aspects of COC terminal differentiation may be altered. A comparison of CC transcriptomes obtained from *in vitro* matured COCs with those obtained from *in vivo* counterparts highlighted critical deficiencies affecting several cumulus molecular pathways known to support developmental potential of the oocyte in mice, humans and cattle ( Brown *et al*., 2017 ). The expression of genes involved in several CC functions such as epidermal growth factor (EGF)-like signalling, extracellular matrix production, glucose metabolism, fatty acid metabolism and immune-like processes, were seen to be impaired during the *in vitro* procedure. The alteration of terminal molecular events in CCs before fertilisation could compromise cumulus-oocyte dialogue and hence the full development of oocyte competence. Studies using a bovine model have shown that the rise in PTGS2-related PGE2 production that occurs in CCs concomitantly with the resumption of meiosis is affected by IVM conditions and the presence of exogenous EGF ( Nuttinck *et al*., 2008 ). However, PTGS2 expression remains weaker than that which is observed after *in vivo* maturation ( Nuttinck *et al*., 2002 ). Several aspects of terminal differentiation of the bovine COC are affected by the level of PGE2 present in the oocyte environment during IVM ( Nuttinck *et al*., 2011 ). The inhibition of cumulus PGE2 production reduces the kinetic of meiosis progression, oocyte MAPK activation and cumulus expansion. Recent publications have recommended the supplementation of IVM media with bioactive molecules that are involved in cumulus-oocyte interplay in order to counterbalance the alterations induced by the *in vitro* procedure ( Richani and Gilchrist, 2018 ). We previously reported that the addition of PGE2 to IVM/IVF media promotes embryonic cell survival and cell lineage development during the two weeks of the preimplantation period in cattle ( Nuttinck *et al*., 2017 ).

## Conclusion

Disruption or deregulation of the interactions between a maturing oocyte and surrounding CCs can affect the final stages of the storage of maternal factors and consequently of subsequent embryonic development. One of the most promising options to improve the viability of *in vitro* produced embryos is to optimise the *in vitro* maturation conditions in order to preserve the integrity of cumulus-oocyte coupling, which will contribute to achieving the full developmental competence of the oocyte.
